# The Alzheimer's disease β-secretase enzyme, BACE1

**DOI:** 10.1186/1750-1326-2-22

**Published:** 2007-11-15

**Authors:** Sarah L Cole, Robert Vassar

**Affiliations:** 1 Department of Cell and Molecular Biology, The Feinberg School of Medicine, Northwestern University, Chicago Avenue, Chicago, IL, USA

## Abstract

The pathogenesis of Alzheimer's disease is highly complex. While several pathologies characterize this disease, amyloid plaques, composed of the β-amyloid peptide are hallmark neuropathological lesions in Alzheimer's disease brain. Indeed, a wealth of evidence suggests that β-amyloid is central to the pathophysiology of AD and is likely to play an early role in this intractable neurodegenerative disorder. The BACE1 enzyme is essential for the generation of β-amyloid. BACE1 knockout mice do not produce β-amyloid and are free from Alzheimer's associated pathologies including neuronal loss and certain memory deficits. The fact that BACE1 initiates the formation of β-amyloid, and the observation that BACE1 levels are elevated in this disease provide direct and compelling reasons to develop therapies directed at BACE1 inhibition thus reducing β-amyloid and its associated toxicities. However, new data indicates that complete abolishment of BACE1 may be associated with specific behavioral and physiological alterations. Recently a number of non-APP BACE1 substrates have been identified. It is plausible that failure to process certain BACE1 substrates may underlie some of the reported abnormalities in the BACE1-deficient mice. Here we review BACE1 biology, covering aspects ranging from the initial identification and characterization of this enzyme to recent data detailing the apparent dysregulation of BACE1 in Alzheimer's disease. We pay special attention to the putative function of BACE1 during healthy conditions and discuss in detail the relationship that exists between key risk factors for AD, such as vascular disease (and downstream cellular consequences), and the pathogenic alterations in BACE1 that are observed in the diseased state.

## Background

AD is the most prevalent form of dementia, and current indications show that twenty-nine million people live with AD worldwide, a figure expected rise exponentially over the coming decades. It has been recently estimated that the worldwide costs for dementia care are $315.4 billion annually (US; Alzheimer's Association). Clearly, blocking disease progression or, in the best-case scenario, preventing AD altogether would be of benefit in both social and economic terms. However, current AD therapies are merely palliative and only temporarily slow cognitive decline, and treatments that address the underlying pathologic mechanisms of AD are completely lacking. While familial AD (FAD) is caused by autosomal dominant mutations in either amyloid precursor protein (APP) [1,2] or the presenilin (PS1, PS2) [3,4] genes, the underlying cause (s) of the remaining ~98% of so-called sporadic AD (SAD) cases remain elusive. However, specific risk factors for AD have been recently identified and include aging, the presence of the apolipoprotein E4 (ApoE4) allele [5] and vascular diseases such as stroke and heart disease [6-10].

### AD pathology

Pathologically, AD is characterized by the accumulation of amyloid beta peptide (Aβ), as fibrillar plaques and soluble oligomers in high-order association brain regions. The presence of intracellular neurofibrillary tangles, neuroinflammation, neuronal dysfunction and death further characterizes this disease. Mounting evidence suggests that Aβ plays a critical early role in AD pathogenesis, and the basic tenant of the amyloid (or Aβ cascade) hypothesis is that Aβ aggregates trigger a complex pathological cascade which leads to neurodegeneration [11]. A strong genetic correlation exists between FAD and the 42 amino acid Aβ form (Aβ42; reviewed in [12-14]). Aβ is derived from APP and mutations in APP and PS increase Aβ42 production and cause FAD with nearly 100% penetrance. Down's syndrome (DS) patients, who have an extra copy of the APP gene on chromosome 21, and FAD families with a duplicated APP gene locus [15], exhibit total Aβ overproduction and all develop early-onset AD. In FAD, the Aβ42 increase is present years before AD symptoms arise, suggesting that Aβ42 is likely to initiate AD pathophysiology. The robust association of Aβ42 overproduction with FAD argues strongly in favor of a critical role for Aβ42 in the etiology of AD, including in SAD. Fibrillar and oligomeric forms of Aβ appear neurotoxic in vitro and in vivo. Importantly, in specific transgenic (Tg) mouse models of AD the lack of Aβ correlates with the absence of neuronal loss and improved cognitive function [16-18]. Such data provides direct evidence for the amyloid hypothesis in vivo, and also indicates that Aβ is directly responsible for neuronal death. Consequently, strategies to lower Aβ42 levels in the brain are anticipated to be of therapeutic benefit in AD.

### Aβ genesis

**Figure 1:**
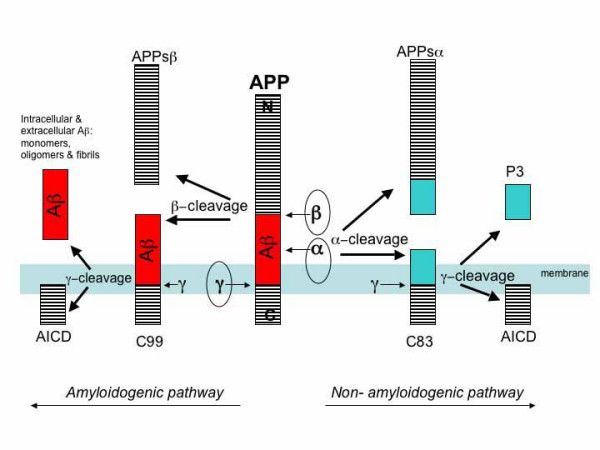
**APP metabolism by the secretase enzymes**. APP is sequentially cleaved by BACE1, the β-secretase, and γ-secretase, a complex comprised of presenilin, nicastin, Aph1 and Pen2, to generate Aβ. BACE1 cleavage of APP is a prerequisite for Aβ formation and is putatively the rate-limiting step in Aβ genesis. BACE1 cleavage of APP forms the N-terminus of the peptide, and two cleavage fragments are liberated: APPsβ, a secreted ectodomain, and C99, a membrane bound fragment. C99 is the substrate for γ-secretase, and C99 cleavage generates the AICD together with the C-terminus of Aβ. Aβ formation is prevented by the activities of α-secretase, which has been identified as TACE, ADAM9 and ADAM10. α-secretase cleaves APP to generate the secreted ectodomain, APPsα and membrane bound fragment, C83. C83 is subsequently cleaved by the γ-secretase complex to yield the 3 KDa fragment, P3 and the AICD.

Aβ peptide is generated following the sequential cleavage of APP by β- and γ-secretase in the amyloidogenic pathway (reviewed in [19,20]). Aβ genesis may be precluded if APP is cleaved by α-secretase within the Aβ domain in the non-amyloidogenic pathway (Fig. 1). Recently, the secretases have been identified and the β-secretase is known to be β-site APP cleaving enzyme I (BACE1; [21-25]), a novel aspartyl protease. The γ-secretase appears as a complex of proteins consisting of PS1 or PS2 [26,27], nicastrin [28], Aph1 and Pen2 [29,30], whereas three putative α-secretases have been identified as TACE (TNF-α converting enzyme; [31], ADAM (a disintegrin and metalloprotease domain protein)-9 and ADAM-10 [32]. BACE1 cleavage of APP is a pre-requisite for Aβ formation. Aβ genesis is initiated by BACE1 cleavage of APP at the Asp+1 residue of the Aβ sequence to form the N-terminus of the peptide. This scission liberates two cleavage fragments: a secreted APP ectodomain, APPsβ and a membrane-bound carboxyl terminal fragment (CTF), C99. C99 is subsequently cleaved by γ-secretase to generate the C-terminus of the Aβ peptide and an APP intracellular domain (AICD). Interestingly, it has been shown that the AICD may play a role in transcriptional transactivation [33]. Cleavage by the γ-secretase complex is not precise; while the majority of Aβ peptides liberated by γ-secretase activity end at amino acid 40 (Aβ40), a small proportion end at amino acid 42 (Aβ42). It is this γ-secretase-dependent cleavage that is affected by most FAD mutations to cause excess generation of Aβ42 in FAD. In the alternative, non-amyloidogenic pathway, α-secretase cleavage of APP occurs within the Aβ domain at Leu+17. α-secretase cleavage produces the secreted APPsα ectodomain, and a CTF, C83, which in turn is cleaved by γ-secretase to form the non-amyloidogenic 3 kDa fragment, p3. In many instances, an increase in non-amyloidogenic APP metabolism is coupled to a reciprocal decrease in the amyloidogenic processing pathway, and vice-versa, as the α- and β-secretase moieties compete for APP substrate [23,34].

Given that BACE1 is the initiating enzyme in Aβ generation, and putatively rate-limiting, it is considered a prime drug target for lowering cerebral Aβ levels in the treatment and/or prevention of AD.

### BACE1: The β-secretase

Prior to its identification, numerous studies were undertaken to define the characteristics of β-secretase activity. Although the majority of body tissues exhibit β-secretase activity [35], highest activity levels were observed in neural tissue and neuronal cell lines [36]. Indeed, β-secretase appeared to predominate in neurons, with the level of β-secretase activity appearing lower in astrocytes [37]. Data showing that β-secretase efficiently cleaved only membrane-bound substrates [38] indicated that the enzyme was likely membrane-bound or closely associated with a membrane protein. Furthermore, as highest β-secretase activity was detected at acidic pH [28,39-41], within the subcellular compartments of the secretory pathway, including the trans-Golgi network (TGN) and endosomes [42,43], it was predicted that the active site of this enzyme is within the lumen of acidic intracellular compartments.

The sequence preference for β-secretase was determined from site-directed mutagenesis of the amino acids surrounding the cleavage site in APP [38]. Substitutions of larger hydrophobic amino acids (such as Leu found in the Swedish FAD mutation) for the Met residue at P1 improve the efficiency of β-secretase cleavage. Conversely, substitution of the smaller hydrophobic amino acid Val at the same position inhibits cleavage. Many other substitutions at this site and at surrounding positions decrease cleavage, and indicate that the β-secretase is highly sequence-specific. Radiosequencing demonstrated that Aβ isolated from amyloid plaques, as well as that produced in cell lines, predominantly begins at the Asp+1 residue of Aβ [44], although minor Aβ species begin at Val-3, Ile-6, and Glu+11 [35]. Inhibitor studies suggest that the Val-3 and Ile-6 species are generated by a protease that is different from the β-secretase [45]. However, the Glu+11 species is produced in parallel with Asp+1 Aβ [46], suggesting that β-secretase is responsible for cleaving at both these positions. Interestingly, the Glu+11 species is the predominant form of Aβ made in rat primary neuron cultures [46]. Finally, β-secretase activity is insensitive to pepstatin, an inhibitor of many (but not all) aspartic proteases.

During 1999–2000, five teams concluded that the novel transmembrane aspartic protease BACE1 (also named memapsin and Asp2) was the β-secretase [21-25]. Indeed, BACE1 exhibited all the known characteristics of the β-secretase. The 501 amino acid sequence of BACE1 bears the hallmark features of eukaryotic aspartic proteases of the pepsin family. BACE1 has two aspartic protease active site motifs, DTGS (residues 93–96) and DSGT (residues 289–292), and mutation of either aspartic acid renders the enzyme inactive [21,47]. Like other aspartic proteases, BACE1 has an N-terminal signal sequence (residues 1–21) and a pro-peptide domain (residues 22–45) that are removed post-translationally, so the mature enzyme begins at residue Glu46 [47]. Importantly, BACE1 has a single transmembrane domain near its C-terminus (residues 455–480) and a palmitoylated cytoplasmic tail [48]. Thus, BACE1 is a type I membrane protein with a luminal active site, features predicted for β-secretase. The position of the BACE1 active site within the lumen of intracellular compartments provides the correct topological orientation for cleavage of APP at the β-secretase site. As observed with other aspartic proteases, BACE1 has six luminal cysteine residues that form three intramolecular disulfide bonds and several N-linked glycosylation sites [49].

The pattern and level of BACE1 expression is largely consistent with those of β-secretase activity in cells and tissues [23,24,50]. The levels of BACE1 mRNA are highest in brain and pancreas and are significantly lower in most other tissues. Moreover, BACE1 mRNA is highly expressed in neurons but little is found in resting glial cells of the brain, as expected for β-secretase. The protein is abundant in both normal human and AD brain [23,50]. Given the low levels of β-secretase activity in the pancreas, the high pancreatic mRNA expression was initially confusing [22]. However, subsequent reports indicated that BACE1 mRNA transcripts in pancreas largely consist of a splice variant missing the majority of exon 3 [51,52]. This splice variant encodes a BACE1 isoform devoid of β-secretase activity, thus reconciling the paradoxically high BACE1 mRNA levels with the low β-secretase activity found in the pancreas. The functional relevance of this pancreas-specific splice variant remains unclear.

BACE1 induces a dramatic increase in β-secretase activity when transfected into stable APP-overexpressing cell lines. The immediate products of β-secretase cleavage, APPsβ and C99, are increased several fold over levels found in untransfected cells, and Aβ production is also elevated. Interestingly, APPsα levels are reduced upon BACE1 transfection, indicative that α- and β-secretases compete for APP substrate in cells. In contrast to the effects of BACE1 transfection, treatment of APP-overexpressing cells with BACE1 antisense oligonucleotides decreases BACE1 mRNA levels and inhibits β-secretase activity [23,24]. BACE1 antisense inhibition reduces production of APPsβ, C99, and Aβ in cells; conversely, APPsα and C83 generation is elevated.

BACE1 cleaves APP only at the known β-secretase sites of Asp+1 and Glu+11 of Aβ [23]. Moreover, purified recombinant BACE1 directly cleaves APP substrates at these same sites *in vitro*, demonstrating that the BACE1 molecule intrinsically exhibits protease activity [23,24]. The sequence specificity of purified BACE1 is the same as β-secretase. For example, it cleaves Swedish mutant APP substrate much more efficiently than wild type, and does not cleave a P1 Met-Val mutant substrate that is resistant to β-secretase cleavage. Like β-secretase, BACE1 has optimal activity at ~pH 4.5, is resistant to inhibition by pepstatin, and is localized within acidic subcellular compartments of the secretory pathway, primarily the Golgi apparatus, TGN and endosomes. Taken as a whole, the properties of BACE1 correlate very well with the previously established characteristics of β-secretase activity in cells and tissues.

### BACE1 knockouts: The consequences of BACE1 deficiency

Unequivocal proof that BACE1 was the major β-secretase in the brain was provided by data derived from BACE1 knockout (BACE1^-/-^) mice. In addition, BACE1^-/- ^mice have also been used to determine whether BACE1 has any vital function *in vivo*, or if it is dispensable. To investigators interested in the therapeutic development of BACE1 inhibitors such knowledge is of critical importance.

A number of strategies were used to achieve inactivation of the BACE1 gene [53-55], and initial data indicated that the absence of BACE1 expression did not appear to adversely affect embryonic development, or significantly affect the morphology, physiology, biochemistry, and gross behavior of post-natal or adult knockout mice [54,55]. Gross behavioral and neuromuscular parameters were investigated in order to examine brain function in knockout mice [54,55] and it was established that no demonstrable differences existed, as compared to wild-type mice. Overall these initial findings indicated that the absence of BACE1 is well tolerated *in vivo *and did not appear to cause untoward effects in the embryonic, post-natal, or adult mouse.

Data generated from BACE1-deficient mouse models was unanimous in demonstrating that β-secretase activity is abolished in brains and cultured neurons of BACE1^-/- ^mice. Unlike wild-type mice, which produce endogenous Aβ at low levels, Swedish APP-overexpressing Tg mice (Tg2576; [56]) produce robust levels of brain Aβ and during aging develop Aβ plaques in the brain. Importantly, BACE1^-/-^•Tg2576 bigenic mice lacked all forms of brain Aβ, as well as APPsβ and C99, as compared to BACE1^+/-^•Tg2576 or BACE1^+/+^•Tg2576 mice [54]. Thus all products of APP processing by β-secretase, including Aβ, were abolished in BACE1 knockout brain, unequivocally proving that BACE1 is the major, if not only, β-secretase that is absolutely required for Aβ generation in the brain. Findings from *in vitro *studies of BACE1^-/- ^primary neurons and brain tissue added further support for the predominant role of BACE1 in Aβ generation [53,55]. BACE1^-/-^•Tg2576 mice not only lack cerebral Aβ, but also fail to develop amyloid plaques with age [57]. Tg2576 mice begin to deposit amyloid in the brain at ~9–12 months of age. Conversely, BACE1^-/-^•Tg2576 bigenic mice show no evidence of amyloid deposits even at 13 months of age. Similar results were obtained by Laird and colleagues using different BACE1^-/- ^and APP Tg mice [18]. Taken together, these results demonstrate BACE1 is required for amyloid formation.

We recently conducted studies to determine whether BACE1 deficiency, and the consequent ablation of Aβ, is sufficient to rescue memory deficits in Tg2576 mice [16]. In AD brain, both fibrillar and soluble Aβ accumulate [58,59]. We demonstrated that memory deficits and cholinergic dysfunction in the hippocampus did not develop in BACE1^-/-^•Tg2576 bigenic mice that lacked Aβ, while florid deficits were apparent in Aβ-overproducing Tg2576 monogenics. Because the Aβ in Tg2576 at the time of testing was non-fibrillar and soluble, we concluded that soluble Aβ assemblies rather than amyloid plaques are responsible for at least some aspects of AD-related memory deficits. This data further validates BACE1 as a prime therapeutic target for AD, and also provides direct evidence for the amyloid hypothesis *in vivo*.

APP/PS1 double Tg mice exhibit accelerated Aβ accumulation and AD-associated memory deficits as compared to single Tg mice. These bigenic animals have been examined to determine the consequences of BACE1 ablation in aged animals. Aged APPswe;PS1ΔE9 Tg mice display abundant amyloid pathology and exhibit impairments in water maze learning [18]. However, APPswe;PS1ΔE9 lacking BACE1 performed as well as wild type controls [18]. Amyloid pathology was not detected in the APPswe;PS1ΔE9;BACE1^-/- ^mice, thus demonstrating that BACE1 deletion abolishes amyloid deposition and prevented spatial reference memory deficits in aged APPswe;PS1ΔE9 mice. We observed a similar scenario in the 5XFAD APP/PS1 Tg mouse model [17,60,61], in which an APP transgene carrying the Swedish (K670N, M671L), London (V717I) and Florida (I716V) mutations is co-expressed with a PS1 transgene carrying double FAD mutations (M146L and L286V; [60]). 5XFAD mice exhibit aggressive pathology with plaque deposition occurring at 2 months and exhibit significant neuronal loss in AD-sensitive brain regions [60]. We clearly demonstrated that deficits in hippocampus-dependent temporal associative learning found in 5XFAD mice were rescued in BACE1^-/-^;5XFAD mice [61] reviewed in [62]. Not only were the elevated Aβ levels observed in 5XFAD mice ablated in BACE1^-/-^;5XFAD bigenic mouse brain, but the genetic abrogation of BACE1 also prevented neuronal loss [17]. These data provide strong support, at least in Tg AD models, for the role of Aβ peptides in age-associated cognitive impairments, and also indicate that Aβ is ultimately responsible for neuronal death.

While initial studies of BACE1 knockout mice did not reveal gross alterations in behavior, recently, more precise behavioral phenotyping studies of BACE1^-/- ^mice have revealed abnormalities in cognitive and emotional functions, suggesting potential mechanism-based toxicities resulting from complete BACE1 inhibition [16,18,61]. We reported that BACE1^-/- ^mice were impaired in both spatial and reference memories. Further, these mice also exhibited impairments in temporal associative memory although they appeared normal in social recognition. These data raise the possibility that BACE1 is required for some normal hippocampal memory processes [16,61]. Consistent with our findings, others have also reported that mice deficient in BACE1 exhibited impaired spatial reference and working memories [18,63].

BACE1 is crucial for Aβ generation, and the normal production of Aβ in the brain raises the possibility that rather than being a toxic by-product of APP metabolism, Aβ may fulfill a regular physiological function. Indeed, the data described above, indicating that complete abrogation of Aβ was associated with impaired memory performance, suggests a role for Aβ in normal memory [16]. These data were consistent with earlier reports of a potential physiological role of Aβ in normal neuronal function [64,65], and a recent report suggests that the production of endogenous Aβ is an important physiological regulator of potassium channel expression and negatively modulates neuronal excitability [66]. However, our data indicate that BACE1 deficiency does not impact all types of hippocampal learning [16] and it is clear that further work is required to examine the putative normal role of Aβ *in vivo*, under non-pathological conditions. Alternatively, BACE1 cleavage of APP leading to AICD-mediated gene transcription may be important for cognitive function [67].

When suitable BACE1 inhibitors are developed, it will be almost impossible to completely suppress BACE1 enzymatic activity in vivo. Interestingly, Singer and colleagues used RNA interference (RNAi) to silence BACE1 and demonstrated that a partial reduction in BACE1 can improve amyloid neuropathology including the deposition of Aβ, alongside cognitive deficits in APP Tg mice [68]. Furthermore, comparison of BACE1 homozygous and heterozygous (BACE1^+/-^) knockout mice indicated that BACE1^+/- ^mice exhibited normal spatial memory function compared to BACE1^-/- ^mice, suggesting that partial inhibition of BACE1 may not affect normal learning and memory processes. Laird and colleagues demonstrated that Aβ burden appears sensitive to BACE1 dosage in young animals, with Aβ levels reduced to ~60–70% in APPswe;PS1ΔE9; BACE1^+/- ^compared to age-matched APPswe;PS1ΔE9 animals [18]. However, the Aβ burden was not altered in older mice, despite the same decrease in BACE1 levels, indicating that BACE1 is no longer a limiting factor in the aged mouse. Consistent with these findings was that older APPswe;PS1ΔE9; BACE1^+/- ^mice were significantly impaired in the Morris water maze, indicating that 50% reductions in BACE are not sufficient to significantly ameliorate cognitive deficits in aged mouse in contrast to complete BACE1 ablation. Although further work is required, these data indicate that a partial suppression of BACE1 may have the most benefit for the earlier phases of Aβ-dependent cognitive impairments. However, the age-dependent benefits of partial reduction BACE1 expression appear complex. McConlogue and colleagues recently reported that, in contrast to the observations of Laird, a 50% decrease in BACE1 levels exerts little impact on Aβ levels in young APP Tg mice, but led to a dramatic reduction in Aβ levels and synaptic deficits in aged mice [69]. The reasons as to why these findings appear discordant with those of the previous study remain to be determined, but may be due to the expression of different APP moieties, APP with the Swedish mutation [18] vs. APP with the V717F mutation [69]. The Swedish mutation enhances the cleavage of APP by BACE1. As McConlogue and colleagues discuss, the co-expression of APPswe with the PS1 transgene in the Laird study led to an aggressive model of plaque development that may somehow impact the sensitivity of APP metabolism to alterations in BACE1 levels.

Together with the putative effect of complete BACE1 ablation on specific cognitive functions, recent findings have indicated that BACE1 deficiency might be associated with other learning-unrelated phenotypes. BACE1^-/- ^mice exhibited a higher mortality rate in early life [70], and those on the PDAPP background displayed severe seizures [63]. In addition, mice deficient in BACE1 appeared hyperactive, with enhanced locomotion [18,70]. BACE1^-/- ^mice spent more time in central parts of the open-field and visited open-arms in the plus-maze test more often than wild type controls, indicating that BACE1 may play some role in anxiety [18]. However, the putative role that BACE1 may play in emotion appears complicated. Indeed, in contrast to Laird [18], Harrison and colleagues previously reported that BACE1^-/- ^mice showed timid, more anxious behavior [71]. While strain differences may account for these apparently contrasting data, the exact reasons underlying these putative opposing effects of BACE1 on emotion remain to be determined.

In conclusion, the lack of Aβ generation in the brains of BACE1 deficient mice indicates that therapeutic inhibition of BACE1 should reduce cerebral Aβ levels and amyloid development, an outcome likely to be beneficial for AD. While recent studies indicate that complete blockage of BACE1 activity may be associated with certain undesirable side-effects (also see "Putative Non-APP BACE1 Substrates" section below), important data demonstrates that in specific AD Tg models, partial reduction of BACE1 levels may improve cognitive deficits and amyloid neuropathology including Aβ deposition.

### BACE2, the BACE1 homologue

BACE2 is a homologue of BACE1 that is mapped to the DS critical region on chromosome 21 [72]. The amino acid sequences of BACE1 and BACE2 are ~45% identical and 75% homologous [73,74]. As with BACE1, the BACE2 C-terminal domain is significantly larger than other aspartic proteases, although overall the BACE2 structure contains features typical of this protease family. Unlike BACE1, pro-BACE2 requires autocatalytic pro-domain processing for enzymatic activation [75]. BACE1 and BACE2 have distinct transcriptional regulation and function [73]. BACE2 mRNA has been observed at low levels in most human peripheral tissues. However, unlike BACE1, which is enriched in neuronal populations [23], human adult and fetal whole brain express very low or undetectable levels of BACE2 mRNA [50,74]. *In vitro*, BACE2 can cleave APP at the β-secretase cleavage site [76,77] and BACE2 appears to be primarily responsible for Aβ production in Flemish mutant APP transfected cells [76]. However, other studies have demonstrated that BACE2 functions as an alternative α-secretase and as an antagonist of BACE1 [78,79]. BACE2 does not get upregulated to compensate for a lack of BACE1 in knockout mice [57]. Further evidence that BACE2 functions not as a β-secretase comes from a recent study which identifies BACE2 as a novel theta (θ)-secretase. Radiosequencing clearly demonstrated that the major BACE2 cleavage site is between Phe+19 and Phe+20 sites of APP, thus BACE2 cleaves APP at a novel θ-site downstream of the α-secretase cleavage site [80]. Cleavage of APP by BACE2 at this site abolishes Aβ production. Furthermore, overexpression of BACE2 reduced Aβ production in primary neuronal cultures derived from APP Tg mice [80].

### BACE1 expression

Increasingly, reports indicate that BACE1 expression is tightly regulated at both the transcriptional and translational level (reviewed in [81]). Insight into the regulation of BACE1 gene expression may aid identification of mechanisms that lead to disease, illuminate the role of BACE1 in normal biology, and may suggest approaches to inhibit BACE1 therapeutically. To this end, both the human and rat BACE1 gene promoters have been sequenced and analyzed [82-85]. The BACE1 gene spans ~30 kilobases (kb) on human chromosome 11q23.2 and includes 9 exons. The BACE1 gene promoter lacks the typical CAAT and TATA boxes, but contains six unique functional domains and three structural domains of increasing sequence complexity as the ATG start codon is approached [86]. It also contains a variety of transcription factor binding sites, including those for Sp1, GATA-1, AP1, AP2, CREB, estrogen and glucocorticoid receptors, NFκB, STAT1, HIF-1 and HSF-1, among others. It is likely that these sites influence transcriptional activity from the BACE1 promoter, and it is interesting to note that a number of these transcription factor binding sites become activated in response to cell stress.

A strong inflammatory reaction is present in AD brain and long-term NSAID use reduces the risk of AD, suggesting inflammation may play an important role in AD pathophysiology [87]. The BACE1 gene promoter also has a binding site for the transcriptional regulator proliferator-activated receptor γ (PPARγ; [88]). Activation of PPARγ with nonsteroidal anti-inflammatory drugs (NSAIDs) or PPARγ agonists cause repression of BACE1 gene promoter activity, while proinflammatory cytokines that reduce PPARγ levels lead to increased BACE1 mRNA [88]. Thus, the effects of inflammation and NSAIDs on AD may involve, at least in part, the action of PPARγ on BACE1 gene expression.

Neurons are responsible for the major portion of BACE1 and Aβ expression in the brain under normal conditions, and this is also likely to be true during AD. However, evidence is mounting that glia, and astrocytes in particular, may produce significant levels of BACE1 and Aβ, especially during inflammation. Glia out-number neurons by ~10:1, so even a slight increase in glial BACE1 expression might contribute substantially to cerebral Aβ and exacerbate AD pathology. NFκB is increased in both aged and AD brain. Interestingly, whereas NFκB acts as a repressor in neurons, this transcription factor acts as an activator of BACE1 transcription in activated astrocytes present in the central nervous system (CNS) during chronic stress [89], a feature observed in AD. In addition, following exposure to Aβ, a functional NFκB site was stimulated in neural cells suggesting that elevated NFκB in the brain may significantly contribute to increased Aβ levels, acting as a positive feedback loop of chronic inflammation, astrocyte activation, and increased BACE1 transcription. Proinflammatory cytokines such as interferon γ (IFNγ), tumor necrosis factor α (TNFα) and interleukin β (IL1β) have been shown to increase Aβ secretion in cultured human astrocytes and astrocytic cell lines [90], and BACE1 levels rise, at least with IFNγ treatment [91,92]. Injection of INFγ into mouse brain led to elevated astrocytic BACE1 expression. Molecular analysis indicated that INFγ activated JAK2 and ERK1/2. Following phosphorylation, STAT-1 then binds to the putative STAT1 binding sequence in the BACE1 promoter region to modulate astrocytic BACE1 expression [92].

Although previous studies have reported BACE1 immunoreactivity in reactive astrocytes around amyloid plaques in both Tg2576 mice [93] and human AD brain [94-96], subsequent analyses by our group on other APP Tg and AD brains show that plaques elevate BACE1 levels in neurons not astrocytes [97]. This discrepancy likely results from the use of BACE1 antibodies in the earlier reports that were not monospecific for BACE1, while our study used a novel, extensively validated BACE1 antibody, BACE-Cat1, that does not cross-react with other proteins.

Although the BACE1 elevation in AD primarily occurs in neurons, the amplified number of astrocytes in AD brain is likely to result in a significant increase of astrocytic Aβ production. Interestingly, treatments with the NSAID ibuprofen or pioglitazone, a PPARγ agonist, decrease BACE1 and reduce plaque load [98]. Furthermore, BACE1 levels appear to rise at focal sites of glial activation even before plaques begin to develop [99].

While it is apparent that BACE1 expression can be tightly regulated at the transcriptional level, in some studies BACE1 mRNA levels in either neurons or astrocytes around amyloid plaques in APP transgenic brain are not elevated [97,100,101], implying that a post-translational mechanism may be responsible for the BACE1 increase. Increased BACE1 protein in the absence of altered BACE1 mRNA levels might be caused by alterations in BACE1 protein stability and/or changes in the rate of BACE1 translation. Indeed, features of BACE1 5' untranslated region (5'UTR) such as the GC content, the length, evolutionary conservation and the presence of upstream AUGs indicate that this 5'UTR may play an important role in the regulation of translational control and several studies indicate that BACE1 may be regulated in this fashion [102-104].

Overall, the evidence suggests that astrocytes may express significant levels of BACE1 and contribute to Aβ production, at least under certain proinflammatory conditions. In addition to inflammation other conditions may cause BACE1 expression to increase in the brain, including oxidative stress, traumatic brain injury (TBI), hypoxia and ischemia. This topic is discussed in detail below.

### The cell biology of BACE1: Post-translational modifications and intracellular trafficking

**Figure 2:**
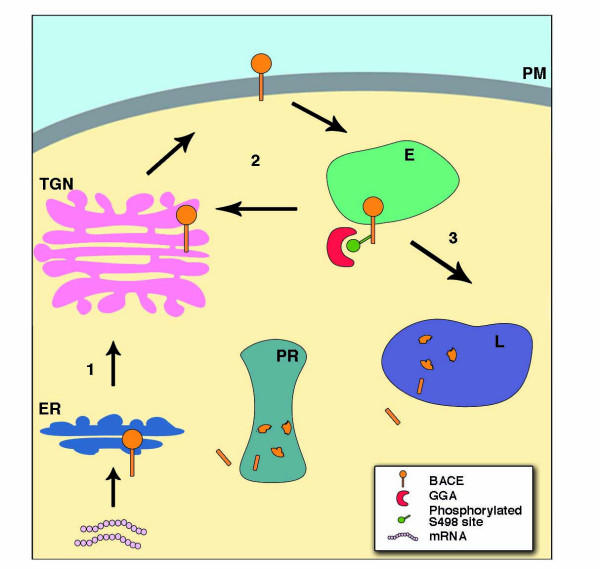
**The intracellular trafficking of BACE1**. (1) Following synthesis in the ER, pro-BACE1 traffics to the Golgi. Here, the propeptide is removed and BACE1 undergoes significant post-translational modifications. The trafficking and localization of BACE1 appear dependent on the ACDL motif, composed of DISLL residues, in its C-terminal tail. Importantly, phosphorylation of the serine 498 residue (S498) is important for binding of BACE1 to the GGA monomeric adaptor proteins which are implicated in the sorting of cargo between the TGN and endosomes and vice versa [127-131], in addition to the Golgi complex to the endosomes [130]. (2) BACE1 traffics to the plasma membrane and while the re-internalization of BACE1 from the cell surface appears to be independent of the phosphorylation state of S498 [131], it has been reported that the di-leucine residues are important for BACE1 endocytosis [126]. Non-phosphorylated BACE1 may recycle from the endosome back to the cell surface [131]. However, BACE1 phosphorylated at the S498 site interacts with GGA1 in the endosome and traffics back to the TGN [131], and (3) the interaction of phosphorylated BACE1 with GGA3 appears to direct BACE1 to the lysosome, where it is degraded [135]. In addition to S498 phosphorylation, the di-leucine residues are also important for the sorting of BACE1 into lysosomal compartments [140]. BACE1 is also degraded in the proteasome [133]. The precise subcellular localization (early verses late endosomes) of the BACE1-GGA complex requires determination, and while it appears likely that BACE1 can traffic from the TGN to the endosome directly, it remains to be demonstrated experimentally.

The cell biology of BACE1 was investigated in order to further understand BACE1 regulation and to identify other potential therapeutic targets in the β-secretase pathway. BACE1 is initially synthesized in the endoplasmic reticulum (ER; Fig. 2) as an immature precursor protein (proBACE1) with a molecular mass of ~60 kDa [49,105-107]. ProBACE1 is short-lived and undergoes rapid maturation into a 70 kDa form in the Golgi, which involves the addition of complex carbohydrates and the removal of the propeptide domain.

BACE1 undergoes extensive post-translational modifications, being glycosylated at four N-linked sites [49]. Mature N-glycosylated BACE1 is sulfated and three cysteine residues within the cytosolic tail of BACE1 become palmitoylated [48]. While glycosylation appears important for enzymatic activity [108], palmitoylation may influence the trafficking and localization of BACE1 [48]. The majority of BACE1 in cells is produced as an integral membrane protein. However, a small fraction of BACE1 undergoes ectodomain shedding, a process that is suppressed by palmitoylation [48]. Inhibition of shedding does not influence processing of APP at the β-site [109]. However, co-expression of APP and the soluble ectodomain of BACE1 in cells increases the generation of Aβ, suggesting the enhanced BACE1 ectodomain shedding may raise amyloidogenic processing of APP [48]. In addition, Murayama and colleagues reported the release of detectable levels of BACE1 holoprotein *in vitro *[110], although the physiological relevance of this event remains to be clarified. Interestingly, active, soluble BACE1 has been detected in human CSF, [111], a finding which raises the potential use of BACE1 detection in an easily accessible biological fluid, such as CSF, in future diagnostic or prognostic applications.

The BACE1 propeptide domain is removed within the Golgi apparatus by cleavage between Arg45 and Glu46 [47,74]. Unlike the majority of aspartic proteases, including BACE2, which cleave the propeptide domain autocatalytically, BACE1 propeptide removal involves intermolecular cleavage by a different protease. Golgi-localized proprotein convertases (PC) are capable of cleaving the BACE1 propeptide and furin, a ubiquitous PC, appears to be the major protease regulating this process [48,74,107]. In contrast to other zymogens, proBACE1 exhibits robust β-secretase activity, indicating that the BACE1 propeptide domain does not significantly suppress protease activity [48,107]. These findings suggest that inhibiting the removal of the BACE1 propeptide would not be an effective therapeutic strategy for reducing Aβ levels in AD. However, proBACE1 may cleave APP early in the biosynthetic pathway leading to the generation of an intracellular pool of Aβ in the ER, thought by some investigators to be particularly neurotoxic [107].

The activity of the BACE1 enzyme is influenced by various post-translational and cell biological events. Mature BACE1 localizes largely within cholesterol-rich lipid rafts [112,113] and replacing the BACE1 transmembrane domain with a glycosylphosphatidylinositol (GPI) anchor exclusively targets BACE1 to lipid rafts and substantially increases Aβ production [114]. Indeed, various types of lipid stimulate BACE1 activity and the raft localization of BACE1 may be enhanced by palmitoylation [115]. Mature BACE1 is relatively stable, having a half-life of over nine hours in cultured cells. Interestingly, ceremide, a lipid with signaling properties, can increase the half-life of BACE1 [116]. BACE1 is capable of forming stable homodimers that exhibit enhanced catalytic activity [117,118]. Interestingly, a variety of molecules have been shown to interact with BACE1 and increase enzyme activity, like prostate apoptosis response-4 (PAR-4) protein [119], while the effects of other interacting partners, such as the copper chaperone for superoxide dismutase-1 [120] and the brain-specific Type II membrane protein BRI3 [121], remain unclear. Conversely, certain molecules have been shown to inhibit the BACE1-APP interaction and thus reduce β-site cleavage, including heparan sulfate [122], reticulon/nogo proteins [123], and sorLA/LR11 [124]. These may provide clues for designing strategies to inhibit BACE1 therapeutically.

Like APP, BACE1 cycles between compartments of the secretory pathway [125-127] and BACE1 activity resides in both the endosomes and secretory pathway. The intracellular trafficking and localization of the BACE1 protein is largely controlled by targeting signals present in the cytosolic portion of the C-terminal tail [127]. The DISLL sequence in the C-terminus of the BACE1 cytosolic tail (amino acids 496–500) is a so-called an acid cluster-dileucine motif (ACDL) and is known to be involved in endosomal trafficking. Deletion of the ACDL motif [125] or mutation of the leucines to alanines [127] alters the subcellular distribution of BACE1, such that a greater proportion of the protein is localized at the cell surface and less is sequestered within endosomes.

The ACDL of BACE1 binds to members of the Golgi-localized γ-ear containing ADP ribosylation factor-binding (GGA) family implicated in the sorting of cargo proteins between TGN and endosomes [128-132]. GGA1, 2 and 3 are monomeric adaptors involved in transport of proteins containing the ACDL motif from the Golgi complex to the endosome, and in the recycling pathway, from endosomes to the TGN [131]. They may also be involved in the delivery of endosomal cargoes to the lysosome [133]. Koh and colleagues recently reported the lysosomal degradation of BACE1, and mutation of the di-leucine motif prevented lysosomal BACE1 accumulation following inhibition of lysosomal hydrolases [134]. Importantly, all three GGA proteins appear to be involved in the trafficking of BACE1 as depletion of any of the three through RNAi caused a significant BACE1 re-distribution [131]. GGA1 interacts with BACE1 and influences BACE1 trafficking through the secretory pathway. The ACDL motif interaction with GGA1 is modulated by serine phosphorylation of the BACE1 motif [126,127,129-131]. BACE1 phosphorylation at the S498 site and interaction with GGA proteins regulate the transport and recycling of the enzyme between TGN, cell surface, and early and late endosomes. Although BACE1 phosphorylation does not appear to dramatically alter β-secretase activity in experimental systems, BACE1 trafficking may have a significant impact on Aβ production in the brain.

Overexpression of GGA1 led to an increase in the levels of both immature BACE1 and APP species [135]. Despite the immature status of BACE1, APP metabolism still occurred and elevations in C99 and APPsβ were observed. However, levels of Aβ were reduced, data indicative that GGA1 blocked APP β-cleavage products from becoming γ-secretase substrates. It was demonstrated that GGA1 confined APP to the Golgi [135]. Thus not only does GGA1 interact with BACE1, but it acts also as a sorting protein that affects APP trafficking and ultimately the proteolysis of this molecule. Interestingly, a very recent report has highlighted the individual roles of the GGA proteins in mediating BACE1 trafficking. In contrast to GGA1, Tesco and colleagues demonstrated that an inhibition of GGA3, via RNAi, led to an elevation of BACE, C99 and Aβ [136]. Caspase cleavage of GGA3 during apoptosis also caused BACE1 levels to increase, suggesting a potential mechanism for BACE1 elevation.

In cultured polarized cells such as neurons or Madin-Darby canine kidney cells, BACE1 is predominantly transported to the axonal/apical compartment, while APP and α-secretase are sorted mainly to the somatodendritic/basolateral compartment [137]. This trafficking pattern is consistent with observations *in vivo *indicating that BACE1 is transported down axons of the perforant pathway [138] and that axon terminals may be major sites of Aβ production [138,139].

While BACE1 has a relatively long half-life, the enzyme is known to be degraded by at least three mechanisms: 1. endoproteolysis within its catalytic domain [140]; 2. the ubiquitin-proteasomal pathway [141]; 3. the lysosomal pathway [134].

### The x-ray structure of BACE1

Structural information about the interaction of substrate with the active site of BACE1 would greatly facilitate the rational design of small molecule BACE1 inhibitors. Towards this end, Sauder et al. [142] used molecular modeling to simulate the BACE1 active site bound with wild-type or mutant APP substrates. The basic structure of most aspartic protease active sites is well conserved and the X-ray structure of pepsin was used to model BACE1. X-ray structural information of a peptide inhibitor bound to rhizopuspepsin was also incorporated to model the interaction with APP. The molecular modeling identified several residues in BACE1 that potentially contribute to substrate specificity. In particular, Arg296 forms a salt-bridge with the P1' Asp+1 residue of the β-secretase cleavage site, thus explaining the unusual preference of BACE1 among aspartic proteases for substrates that are negatively charged at this position. In addition, several hydrophobic residues in BACE1 form a pocket for the hydrophobic P1 residue. The model also showed that the Swedish FAD mutation, LysMet→AsnLeu at P2-P1, interacts more favorably with Arg296 and the hydrophobic pocket of BACE1 than does wild-type substrate, providing an explanation for the enhanced cleavage of this mutation. Conversely, the substitution of Met→Val at P1 blocks the catalytic Asp93 residue, explaining the lack of cleavage of this mutation by BACE1.

Shortly after the molecular modeling study, the X-ray structure of the BACE1 protease domain co-crystallized with a transition-state inhibitor was determined to 1.9 angstrom resolution [143]. As expected, the BACE1 catalytic domain is similar in structure to pepsin and other aspartic proteases, despite the relatively low sequence similarity. Interestingly, the BACE1 active site is more open and less hydrophobic than that of other aspartic proteases. Four hydrogen bonds from the catalytic aspartic acid residues (Asp93 and Asp289) and ten additional hydrogen bonds from various residues in the active site are made with the inhibitor, most of which are conserved in other aspartic proteases. The X-ray structure indicates that Arg296 and the hydrophobic pocket of the active site play an important role in substrate binding, confirming the results of the molecular modeling study. In addition, the bound inhibitor has an unusual kinked conformation from P2' to P4'. The BACE1 X-ray structure suggests that small molecules targeting Arg296 and the hydrophobic pocket residues should inhibit β-secretase cleavage. Moreover, mimicking the unique P2'-P4' conformation of the bound inhibitor may increase the selectivity of inhibitors for BACE1 over BACE2 and the other aspartic proteases.

### Putative non-APP BACE1 substrates

Given the fact that the majority of APP molecules are cleaved by α-secretase moieties, with only a small fraction being BACE1 substrate, it is highly likely that other BACE1 substrates exist. In addition to APP, it is known that BACE1 also cleaves the APP homologues, amyloid precursor-like proteins, APLP1 and APLP2 [144,145]. While the Aβ sequence is absent in both APLP1 and APLP2, relatively little else is known about APLP1 and -2, although it is known that the APLPs can be processed by γ-secretase generating intracellular fragments with potential transcriptional activity [146,147].

While the essential cellular functions of BACE1 under normal conditions have proved somewhat elusive, recent findings have started to define a physiological function for this enzyme. As discussed earlier, given the apparent phenotypic alterations observed in BACE1 deficient mice, the identification of other BACE1 substrates and an understanding of their biological function is essential.

BACE1 is enriched in neuronal populations, and β-secretase processing of APP is modulated by the interaction of BACE1 with neurite growth inhibitor NOGO, a member of the reticulon protein family and a component of myelin [123]. Recently, a role for BACE1 in axonal growth and brain development has been proposed, whereby BACE1 regulates the myelination process [148,149]. The neuronal type III isoform of the epidermal growth factor (EGF)-like factor neuregulin 1 (NRG1) regulates myelination and is a known initiator of peripheral nervous system (PNS) myelination and a modulator of myelin sheath thickness in both the CNS and PNS. BACE1 is transported to axons by a kinesin-1-dependent pathway [150] and the highest levels of BACE1 expression are observed when myelination occurs during the early postnatal stages [149]. Co-expression of BACE1 with type III NRG1 was observed within sensory and motor neurons whose axons project within peripheral nerves. Importantly, data generated from BACE1^-/- ^mice showed that the absence of BACE1 was associated with hypomyelination of both central and peripheral nervous system axons.

In line with previous observations indicating that BACE1 deficient mice exhibit altered synaptic plasticity and decreased cognitive function [16,18,61] reviewed in [62], specific neurological impairments were also associated with the genetic ablation of BACE1 in this myelination study [148]. When compared to wild type brain, BACE1^-/- ^accumulated full length, uncleaved NRG1 and exhibited reduced levels of NRG1 cleavage fragments, findings consistent with a role for BACE1 in the proteolysis of NRG1 [148,149]. It remains undetermined as to whether NRG1 cleavage is required for maintenance of the mature myelin sheath. It should be noted that complete deletion of BACE1 was necessary in order to alter the signaling events and cause hypomyelination. In agreement with previous findings that partial BACE1 inhibition may be without effect on normal learning and memory processes, [18] reviewed in [62], BACE^+/- ^mice did not display any of the biochemical and morphological features observed in the BACE1 null mice.

Synaptic dysfunction precedes overt neurodegeneration during AD progression, and data indicates that BACE1 cleaves APP to generate Aβ in the synaptic terminal [138,139]. Indeed a role for BACE1 in the maintenance of synaptic function has been proposed, and several additional, non-APP BACE1 substrates have recently been identified, some of which may be localized at the terminal, suggesting that BACE1 cleavage of particular substrates may be required for normal function at the synapse.

Voltage-gated sodium channels (VGSC [151]; Na_v_1 [152]) are abundant ion channels responsible for the initiation and propagation of action potentials. These channels are large complexes consisting of an α subunit and at least one β subunit. The VGSCβ subunits are important auxiliary units, although they are not essential for the basic functioning of the VGSC. However, expression of both subunit types is required for full functionality of the VGSC, as well as for the modification of channel properties and intracellular localization. In a manner analogous to APP processing, VGSCβ subunits are substrates of both BACE1 and γ-secretase. BACE1 cleavage generates a membrane-tethered β-CTF [151,152], and the VGSCβ subunits are processed further by γ-secretase, which generates an β2-intracellular domain (β2-ICD; [152]). Recently, the functional ramifications of these cleavage events have been elucidated [152]. β2-ICD regulates expression of the α subunit, Na_v _1.1. Importantly, the increased pool of Na_v_1.1 is maintained within the cell and the BACE1 cleavage of the β2 subunit actually leads to loss of functional membrane channels, a reduction in Na^+ ^current and alterations in membrane excitability [152]. Importantly, the processing of VGSCβ2 and VGSCβ4 by BACE1 has been demonstrated *in vivo *and elevated β2-CTF and Na_v _1.1 were observed in human AD brain tissue [151,152]. Neural activity regulates Aβ production through β- and γ-secretase and synaptic transmission and neuronal activity is depressed by Aβ [65,153]. It is thus plausible that the turnover of membrane-localized functional sodium channels by the sequential processing by BACE1 and γ-secretase in wild type neurons may play a role in such a feedback mechanism as to modulate neuronal activity and endogenous Aβ production.

The proteolytic processing of membrane proteins to their soluble counterparts during ectodomain shedding is an important step in regulating the biological activity of membrane proteins. Ectodomain shedding is carried out by members of the ADAM family and matrix metalloproteases, and to a lesser extent by BACE1 and BACE2 (reviewed in [154]). Ectodomain shedding is the first cleavage event in a two-step proteolytic cleavage event known as regulated intramembrane proteolysis (RIP). Following shedding, the resulting membrane bound CTF, undergoes a second cleavage event within its transmembrane domain, called intramembrane proteolysis. APP undergoes RIP by the α- and β-secretases, with the subsequent intramembrane proteolysis being catalyzed by the γ-secretase complex. Given the fact that APP is only cleaved to a small extent by BACE1, it appeared highly likely that other BACE1 substrates may be found among proteins undergoing ectodomain shedding.

This notion has been recently confirmed by the identification of the lipoprotein receptor-related protein (LRP) as another putative BACE1 substrate. As detailed below, accumulating evidence indicates that elevated cholesterol might be closely involved in AD development. LRP is a type I integral membrane protein which functions as a multifunctional endocytic receptor implicated as having signaling roles in neurons [155]. LRP appears to be intimately associated with AD pathology and previous reports have implicated LRP in the mediation of endocytosis of a number of important AD-linked ligands including APP and ApoE. Indeed, it has been shown that LRP regulates Aβ trafficking, binds Aβ complexes and mediates its degradation. *In vivo*, the absence of LRP in the presence of APP overexpression led to a two fold increase in amyloid deposition, findings supporting the notion that the LRP might play an integral role in Aβ clearance and might be neuroprotective against Aβ toxicity [156]. Like the VGSCβ subunit, LRP is processed in manner analogous to APP, at least *in vitro*. Not only is LRP proteolyzed by matrix metalloproteases, but the γ-secretase cleavage of LRP enables release of the LRP-intracellular domain (LRP-ICD). Furthermore, LRP may be a substrate for BACE1 cleavage [157]. Endogenous BACE1 and LRP co-immunoprecipitate from human brain and it appears as if the LRP-BACE1 complexes occur in lipid-rafts, with the closest association being at the cell surface. Further *in vitro *analyses demonstrated that endogenous levels of BACE1 activity facilitated an increase in the secretion of LRP, in addition to the formation of the LRP CTF. Moreover, increased BACE1 expression facilitated an enhancement of γ-secretase LRP cleavage and release of the LRP-ICD. Indeed, it was also shown that LRP competes with APP for γ-secretase activity [158]. The LRP-ICD has been shown to translocate to the nucleus and interact with Fe65 and Tip60, although whether this occurs under physiological conditions remains to be determined. However, it should be noted that the processing of LRP by BACE1 *in vivo *remains to be determined and data from BACE1 knockouts regarding this matter should prove to be highly informative.

As discussed earlier, neuroinflammation is a pathological feature of AD and increasing evidence suggests that neurotoxicity is mediated by CNS inflammatory processes whereby Aβ is involved in the activation of microglia facilitating the subsequent release of inflammatory cytokines including IL-1β, IL-6 and TNF-α, among others (reviewed in [159]). Interestingly, several putative BACE1 substrates are closely associated with the inflammatory response.

P-selectin glycoprotein ligand 1 (PSGL-1) modulates leukocyte adhesion in inflammatory reactions. Interestingly, cleavage of PSGL-1 by BACE1 liberated cleavage products observed *in vivo*, and no PSGL-1 cleavage fragments were detected in primary cells derived from BACE1 deficient mice, adding further support for the role of BACE1 in PSGL-1 proteolysis [160].

Beta-galactoside alpha 2, 6-sialyltransferase (ST6Gal1) is a second membrane protein involved in regulating the immune response that also appears to be a BACE1 substrate. BACE1 is enriched in neuronal Golgi membranes and ST6GalI is a Golgi-resident sialyltransferase that is secreted out of the cell after proteolytic cleavage. BACE1 and ST6Gal I co-localize in the Golgi and BACE1 overexpression elevates ST6Gal I secretion [161]. Moreover, data from mouse models demonstrated that BACE1 cleaves ST6Gal I *in vivo *[162].

Ectodomain shedding is important in the inflammatory response. The interleukin-1 receptor II (IL-1R2) undergoes shedding and functions as a decoy receptor thought to limit the detrimental effects of IL-1 in the brain. Increased proteolytic processing and secretion of IL-1R2 has been linked to AD pathogenesis [163], and a recent report indicates that all three secretase moieties appear to cleave IL-1R2 [164]. Interestingly, the secretion of IL-1R2 was elevated by both BACE1 and BACE2 overexpression. Cleavage of IL-1R2 by both BACE enzymes occurred at sites that agreed well with the known cleavage specificity of BACE1 and BACE2 and led to the generation of CTF that were similar to those generated by α-secretase cleavage. In contrast to other BACE substrates, including APP, PSGL-1, ST6GalI, VGSCβ and NRG1, the cleavage of IL-1R2 was not reduced in BACE deficient cells. Arguing against the fact that IL-1R2 may not be a physiological substrate of BACE, Kuhn and colleagues demonstrated that BACE1 and BACE2 cleaved IL-1R2 only 4 amino acids away from the α-secretase cleavage site, so close that the α-secretase and BACE IL-1R2 CTFs would be very similar in size and likely impossible to identify individually. Thus, it was suggested that a decrease in BACE IL-1R2 cleavage would be compensated for by an increase in α-secretase cleavage and thus no net change in total IL-1R2 would be observed. Indeed, if BACE cleavage occurs close to the cleavage sites of specific substrates by other proteases, it may be particularly challenging to unequivocally identify a specific protein as a novel BACE substrate.

It has been previously suggested that BACE1 exerts a generalized role in the secretion of membrane proteins, as the BACE1 substrates identified to date are all membrane localized. However, Kuhn and colleagues investigated BACE cleavage specificity and observed that BACE1 and BACE2 did not cleave a number of membrane proteins including TNFα, P-selectin and CD14, suggesting that the proteases did not simply contribute to general membrane protein turnover [164].

### BACE1 dysregulation in AD

Aβ plays a central role in AD pathogenesis. With age, Aβ levels increase and excessive Aβ deposition occurs in AD. While Aβ deposition can be attributed to excessive Aβ production mediated by APP/PS1 mutations or APP gene dosage effects in both FAD and DS, the mechanism by which excessive Aβ accumulation occurs in SAD remains unclear. Reduced Aβ clearance and/or degradation is one potential mechanism leading to increased cerebral Aβ levels in AD. However, it is also possible that small increases in Aβ production over time may tip the balance toward Aβ accumulation. BACE1 is critical for Aβ biosynthesis and it is likely that factors that elevate BACE1 may lead to increased Aβ generation and promote AD. Indeed, FAD cases caused by the APP Swedish mutation, which enhances cleavage by BACE1, imply that increased BACE activity may be sufficient to induce AD pathogenesis. Recently, several important reports have indicated that BACE1 dysregulation maybe involved in AD pathogenesis.

An age-related increase in BACE1 activity in mouse, monkey and non-demented human brain has been reported [165] and there was a positive correlation between elevated BACE1 activity and increased Aβ levels in mouse and human brain regions. Furthermore, significant increases in BACE1 protein and activity have been observed in the AD brain. In high-order association brain regions affected by Aβ deposition BACE1 protein levels and activity were increased significantly in AD brain compared to non-demented control brain [166-170]. While contrasting with other findings [166-170], Li and colleagues reported that the observed elevation of BACE1 activity is correlated with brain Aβ 1-x and Aβ1-42 production in the frontal cortex [170], suggesting that indeed, BACE1 elevation may lead to enhanced Aβ production and deposition. Interestingly, a significant correlation between BACE1 levels and plaque load in AD brains was observed [169,170].

Neuronal loss is a key feature of late-stage AD and so the normalization of protein level and activity measurements to levels of neuronal markers is crucially important. Interestingly, the measures of BACE1 protein and activity levels were even more pronounced in AD brain when normalized to the synaptic marker, synaptophysin [166]. While a separate study indicated that total BACE1 levels in AD temporal cortex were not elevated [171], the ratio of BACE1 protein to specific neuronal markers was significantly increased, indicating that the surviving neurons in AD brain may express higher BACE1 levels than those observed in neurons from control brain.

Interestingly, BACE1 increases have also been observed in DS. People with DS inevitably develop characteristic AD neuropathology thought to result from the extra copy of chromosome 21. However, APP gene dosage alone may not fully account for the AD pathology in DS [172]. Recently, a novel molecular mechanism by which AD may develop in DS has been proposed [173]. Analysis of cerebral cortex derived from DS and normal fetus indicated an elevation of C99, Aβ 40 and Aβ42 in the trisomic tissue. The data indicated that the increase in APP level only partially contributed to Aβ over-generation in DS. Furthermore, the DS tissue showed a significant increase in the levels of total BACE1 protein, in particular mature BACE1 [173]. It was demonstrated that predominantly immobile BACE1 proteins accumulated in the Golgi, whereas BACE1 trafficking was unimpeded in control tissue. Importantly, subcellular fractionation of normal and DS brain tissue showed the marked accumulation of mature BACE1 in the Golgi fraction of DS cells. The authors proposed that the higher levels of mature BACE1 in DS tissue result in higher BACE1 activity leading to elevated C99 and Aβ production. The cause of the BACE1 elevation in DS remains to be determined.

Many biochemical parameters deviate from normal in AD and approximately 100 different proteins have deranged levels or abnormal modifications in AD (reviewed in [174]). Indeed it is difficult to determine from postmortem brain whether a specific change is an epiphenomenon in late-stage AD, or whether it is an early event directly involved in pathogenesis. To address this, we recently examined BACE1 levels in two Tg models of AD [97], namely the 5XFAD mouse [60] that develops amyloid plaques at young ages and exhibits significant neuronal loss, and the Tg2576 mouse [56], which develops plaques at older ages and does not show neuronal death. Interestingly, BACE1 was elevated in the brains of both Tg models and AD patients. Importantly, because the BACE1 increase correlated with amyloid pathology in both Tg models and was observed in both the absence (Tg2576) and presence (5XFAD) of significant neuronal loss, we concluded that BACE1 elevation appeared to be associated with amyloid pathology rather than cell death.

As discussed previously, many BACE1 antibodies produce nonspecific backgrounds in immunohistochemistry and bind to numerous non-BACE1 polypeptides on immunoblots [97]. To circumvent these issues we used the mono-specific BACE-Cat1 antibody and observed the neuronal localization of BACE1, with BACE1 immunoreactivity surrounding Aβ42-containing plaque cores in both the Tg and AD brain. While further studies are required, the co-localization of BACE1 immunoreactivity with synaptophysin, but not MAP2, suggested a presynaptic localization for BACE1. These data are in keeping with previous observations [138] and suggest that the BACE1 elevation occurs in presynaptic neuronal structures around neuritic plaques and that Aβ42 may cause the increase.

### Putative cause of BACE1 elevations in AD

AD pathogenesis is highly complex. Perhaps as a consequence of this complexity, many major questions about this disease remain open. Indeed, apparently basic questions including those regarding the initiating events in AD, and whether such events cause elevate Aβ, remain to be determined. Given the number of pathologies that characterize AD, it is highly possible that there are several events that culminate to trigger AD. We know that Aβ elevation occurs early in the disease and plays a central role in AD pathogenesis. More recently, other pertinent questions have come to light and a major subject for current debate is the cause of BACE1 elevation in AD and whether this elevation could be responsible for the initiation of AD pathology.

Indeed, whether the BACE1 elevation in AD promotes Aβ generation and disease progression remains to be determined. However, an AD feedback loop has long been proposed and our data are suggestive of a positive feedback loop, whereby Aβ42 deposition in AD causes BACE1 levels to rise in nearby neurons. Increased Aβ production may follow, initiating a vicious cycle of additional amyloid deposition followed by further elevated BACE1 levels. Interestingly, a recent in vitro study [175] demonstrated that a relatively small increase in BACE1 expression results in sharply elevated Aβ production, until a plateau is reached, whereby further increases in BACE1 expression and activity have no further effect of Aβ genesis.

Given the observation that BACE1 elevation occurs around Aβ42 plaque cores it seems possible that Aβ42 somehow triggers the BACE1 increase. It is well established that Aβ42 is neurotoxic and such toxicity may induce the BACE1 elevation. Indeed, as previously mentioned, the BACE1 promoter contains regions for transcription factors known to be activated in response to cellular stress. Furthermore, proinflammatory cytokines appear to modulate BACE1 expression [91,92] and following Aβ exposure a functional NFkβ in the BACE1 promoter region was stimulated [89]. However, it remains to be determined as to which is the initiating event, Aβ elevation and deposition or increased BACE1 activity. Interestingly, Tamagno and colleagues have recently proposed that Aβ acts via a biphasic neurotoxic mechanism, which is conformation-dependent, with Aβ oligomers inducing oxidative stress while fibrillar Aβ increases BACE1 expression and activity [168]. Nevertheless, whether such a biphasic mode of action occurs in vivo remains to be determined.

Given the complexity of AD pathogenesis it is highly likely that, in addition to Aβ, a number of other factors could impact BACE1 levels. Aging is the strongest risk factor in AD. Given that BACE1 activity increases with age and to an even greater extent in SAD, it is plausible that AD may reflect an exaggeration of age-related changes in BACE1 activity. It is interesting to note that the vast majority of cardiovascular events occur in older people and there appears to be a close relationship between cardiovascular (and cerebrovascular) disease and AD.

Individuals with AD and cerebrovascular pathologies exhibit greater cognitive impairment than those exhibiting either pathology alone [176-180]. Various types of heart disease (for example, atrial fibrillation, congestive heart failure, coronary heart disease, among others) and stroke (ischemic, hemorrhagic) are intimately associated with elevated AD risk (reviewed in [181]). AD patients exhibit more severe atherosclerosis in the cerebral arteries at the base of the brain (the circle of Willis) when compared to age-matched controls [182]. Indeed cerebral blood supply is limited by the vascular narrowing caused by these lesions [183-185] and many vascular-related AD risk factors have an established association with cerebral hypoperfusion. Importantly, there is increasing evidence from epidemiological [186-189] and neuroimaging studies [190-192] which suggests that vascular risk factors, and the ensuing reduced cerebral blood flow (CBF) and chronic brain hypoperfusion (CBH) are key factors in AD development and may even play a causative role in dementia (reviewed in [181,193]). Evidence suggests that the subcellular changes that are crucial to neurodegeneration development are provoked by CBH brought on by the cardio-cerebral damage associated with cardiovascular events (reviewed in [181]). Indeed, CBH is a pre-clinical condition of mild cognitive impairment (MCI), a condition thought to precede AD, and is an accurate indicator for predicting the development of AD [194-198]. Interestingly, an association between impaired functionality of microvessels and unfavorable evolution of cognitive function in AD patients has been recently reported [199].

CBH can cause hypoxia, in addition to ischemic episodes. The later involves both hypoxia and reoxygenation, which represent forms of cellular stress, and such episodes have also been reported to increase AD risk. Interestingly, oxidative stress has been implicated in AD pathology and is characterized by the presence of oxidative stress markers such as 4hydroxy-2-nonenal (HNE) at the early pathological stages of AD [200,201]. In addition, several studies indicate that defective energy metabolism may play a fundamental role in AD pathogenesis. The expression of several enzymes is downregulated in AD brain, implicating the impairment of brain energy metabolism in this disease [202-204]. Data derived from positron emission tomography imaging indicates that glucose utilization is lower in AD brains than in age-matched control brain. Moreover, both patients with MCI and young adults carrying the ApoE4 allele exhibit reduced glucose metabolism, suggesting that insufficient energy metabolism may also be a factor in preclinical AD.

It is likely that multiple cellular events may account for the neurodegeneration observed in the AD brain and some negate the role of Aβ in AD (reviewed in [181]). However, we hypothesize that the downstream cellular consequences of cardiovascular events, which may indeed play an early and possibly causative role in AD, lead to the significant alterations in Aβ metabolism that are central to AD pathogenesis. Given the strong genetic evidence from FAD cases, we maintain that Aβ fulfills an early and crucial role in AD progression.

The direct contribution of vascular factors to AD pathogenesis remains a contentious issue. However, an increasing body of evidence indicates that Aβ may be vasoactive and cause cerebrovascular impairment by exerting effects on both the systemic and cerebral vasculature (reviewed in [184]). Interestingly, Shin and colleagues reported that deposited, but not soluble, Aβ impaired blood flow when deposited in the vasculature [205]. In addition to exerting causative effects on the vasculature, alterations in Aβ levels may occur as a consequence of cardio- and cerebrovascular disease, possibly via a mechanism involving BACE1 elevations. Indeed, alterations in cholesterol homeostasis facilitate amyloidosis and are associated with elevations in BACE1 levels and activity [206-209]. Furthermore, several key downstream cellular consequences of cardiovascular insults and the resulting CBH, such as hypoxia, energy depletion and cellular stress also increase BACE1 levels and activity [210-216]. These data provide a molecular mechanism that could underlie the effect of specific cardiovascular events on increased Aβ production and consequently AD risk elevation.

#### Hypercholesterolemia and BACE1

Hypercholesterolemia can result in atherosclerosis. Indeed, numerous studies suggest that alterations in cholesterol homeostasis contribute to AD etiology by enhancing Aβ generation [217,218], and elevation of brain Aβ levels following enrichment of dietary cholesterol has been reported [206-208]. The molecular mechanisms linking increased dietary cholesterol with enhanced brain Aβ levels remained unclear for some time (reviewed in [219]). Nevertheless, BACE1 activity is enhanced in lipid microdomains [114] and cholesterol-rich microdomains are critically involved in BACE1 cleavage of APP. Lipid raft-localized APP is cleaved by BACE1, whereas APP outside the rafts is a substrate for α-secretase [113].

To explore the putative molecular mechanism(s) underlying the apparent cholesterol sensitivity of Aβ generation further, Ghribi and colleagues examined the effect of a low dose, relatively long-term, cholesterol-enriched diet on neuronal cholesterol levels, BACE1 and Aβ42 in the rabbit hippocampus [209]. In contrast to control animals, the cholesterol-fed animals exhibited increased levels of neuronal cholesterol, and the cholesterol appeared to co-localize with BACE1, an association accompanied by an increase in both the level of BACE1 protein and activity. The increases with BACE1 corresponded with elevations in APPsβ, C99 and Aβ42. These data suggest that prevention of cholesterol accumulation, or indeed cholesterol reduction may represent a possible strategy for reduction of BACE1 over-activation and may have therapeutic implications for AD. Indeed, earlier reports indicated that long-term treatment with cholesterol-lowering drugs (specific statins) may significantly reduce AD risk [217,218], although several later studies failed to replicate these data [220-222]. The potential of statin therapy in AD still hangs in the balance given the recent finding that Atorvastatin therapy appeared to improve the cognitive ability in patients living with mild-to-moderate AD [223].

#### Hypoxia and BACE1

A shortage of oxygen in the body, a condition referred to as hypoxia, is a direct consequence of hypoperfusion and can facilitate AD pathogenesis [214,224]. Oxygen homeostasis is essential for well being and the brain is particularly susceptible to hypoxic conditions. As detailed previously, BACE1 expression is regulated by multiple mechanisms in a complex manner [73,83,84,225]. Interestingly, BACE1 mRNA expression is increased in APP Tg mice maintained under hypoxic conditions, and the hypoxia appeared to potentiate the memory deficits observed in these mice [214].

It is known that the hypoxia-inducible factor 1 (HIF-1), a member of the basic helix-loop-helix transcription factor family, is principally involved in the regulation of oxygen homeostasis [226]. When oxygen supply is limited, HIF-1 binds to an hypoxia-responsive element (HRE) in gene promoters or enhancers and activates a broad range of genes including those involved in energy metabolism and cell death [227]. As observed *in vivo*, hypoxia increased APP metabolism and Aβ production by upregulating BACE1 activity in vitro. A molecular basis for the observed elevation of BACE1 expression during hypoxia has been recently provided by the identification of a functional HRE in the BACE1 gene promoter [214,224]. Indeed, these data may provide a molecular link between vascular factors and AD that is centered around BACE1 elevations.

#### Mitochondrial dysfunction, oxidative stress, energy depletion and BACE1

Mitochondrial dysfunction in neurons can be caused by many cellular insults including transient hypoxia. Indeed, energy-depleted hippocampal neurons unable to cope with prolonged CBH undergo both oxidative and cellular stress (reviewed in [181]). Importantly, several reports document BACE1 as a stress-induced protease and in view of the apparent importance of metabolic dysfunction and amyloidosis in AD, it is noteworthy that BACE1 upregulation has been observed under a variety of experimental conditions likely involving energy disruption and/or mitochondrial stress [136,212-215,228,229]. Indeed, increased BACE1 levels and activity have been reported in both *in vitro *[210-212] and *in vivo *[213,215] under conditions of altered energy metabolism and cellular stress.

In many aspects of AD, particularly at the molecular level, a chicken-and-egg scenario exists: does Aβ cause BACE1 elevation or vice versa? Does Aβ cause cerebrovascular dysfunction or vice versa? It appears likely that in most cases both scenarios might be true and may operate in a vicious circle of events. This appears to likely be the case with Aβ and oxidative stress. While Aβ accumulation may lead to oxidative stress [230] reviewed in [231], it has also been demonstrated that oxidative stress may lead to Aβ accumulation. As previously indicated, HNE has been observed in AD brain [200,201]. Tamagno reported that HNE addition to cultured NT2 neurons facilitated an increase in BACE1 activity [210]. Interestingly, oxidative stress *in vitro *resulted in significant increases in BACE1 promoter activity [212] and Tamagno and colleagues reported an increase in both BACE1 mRNA and protein levels, with increased BACE1 activity, as determined by enhanced Aβ production, being observed following HNE exposure [210,211].

Inhibitors of mitochondrial respiration are generally considered to cause oxidative stress [232,233] and thus the differentiation of energy inhibition from oxidative stress with regards to the pharmacological blockage of mitochondrial energetic processes *in vivo *is practically impossible. Nevertheless, following acute energy inhibition (and/or oxidative stress) *in vivo *[213], we observed a significant elevation in BACE1 protein. This effect was long-lasting, with BACE1 levels remaining elevated for seven days post-injection, and appeared to correspond with a significant increase in cerebral Aβ40 load in treated mice. In agreement with the previous *in vitro *studies, these data suggest that energy inhibition is potentially amyloidogenic. Support for these observations come from the recent findings that mitochondrial respiratory inhibition and oxidative stress increased BACE1 levels [215]. Moreover, as we previously hypothesized [97], evidence in support of a role for Aβ in elevating BACE1 was provided from data showing that the intravitreal application of fibrillar Aβ42 appeared to exert extremely potent effects and caused enhanced BACE1 expression and activity [215].

The mechanism of the energy-induced BACE1 increase *in vivo *is unclear and will likely be challenging to elucidate. While studies have demonstrated increases in BACE1 mRNA level following oxidative stress and hypoxic insults [211,214], our data indicates that a post-translational mechanism may be implicated in the *in vivo *BACE1 elevation during times of energy depletion [213]. BACE1 mRNA levels did not significantly increase in Tg2576 brain following energy inhibition treatment and the BACE1 protein half life is too long to account for the rapid rise in BACE1 levels observed. As previously discussed reports indicate that the 5'UTR of BACE1 mRNA influences translational efficiency [102-104]. Clearly, further, detailed analysis is required to solve this important issue, but full understanding of such cellular mechanisms may shed light on novel therapeutic approaches for AD.

#### Injury and BACE1

The observations that BACE1 levels are also elevated following brain trauma [228] and ischemia [136,229] add further support for the role of BACE1 as a stress response protein. TBI and stroke are significant risk factors for AD [234,235] and can be modeled experimentally [228,229]. Data derived from a rat model of TBI supports a role for BACE1 elevation in the increase of Aβ levels observed in patients with brain trauma [236,237]. Following injury, elevations in BACE1 mRNA, protein and activity were observed in specific, AD-sensitive brain regions [228].

In an experimental stroke model used to study the effects of transient cerebral ischemia, ischemia led to an elevation in BACE1 protein and activity, and BACE1 immunoreactivity was strongly associated with TUNEL staining, a marker of apoptosis [229]. Interestingly, a recent study has revealed a potential molecular mechanism that may underlie the BACE1 increase following ischemic episodes [136]. Whilst ischemia induces apoptosis, the contribution of apoptosis to AD pathogenesis remains unclear although there is increasing evidence for caspase activation in the AD brain (reviewed in [238,239]). However, Aβ levels in both neuronal and non-neuronal populations are elevated during apoptosis and it was reported that BACE1 levels and associated activity were potentiated during apoptosis [136]. Indeed, caspase activation during programmed cell death induced the BACE1 increase via a post-translational stabilization of BACE1 and a significant impairment in BACE1 degradation and turnover. As previously detailed, the GGA adaptor proteins are implicated in the subcellular trafficking of BACE1. Tesco demonstrated that during apoptosis GGA3 is cleaved by activated caspase-3 [136]. In the rat model of ischemia, this reduction in GGA3 levels was co-ordinated with caspase activation and increased BACE1 protein levels. Furthermore, RNAi silencing of GGA3 caused an increase in the level and activity of BACE1 as determined by elevations in C99 and Aβ. Indeed, He et al have previously demonstrated that RNAi-mediated depletion of GGAs significantly increases endosomal BACE1 levels [131]. Degradation of BACE1 occurs, in part at least, in the lysosomal pathway [134] and a role for GGA3 in the targeting of cargo to the lysosome has been previously reported [133]. Thus, Tesco and colleagues suggested that apoptosis, caused by ischemic events, drives GGA3 depletion and results in the stabilization and accumulation of BACE1 leading to elevated enzymatic activity. Importantly, in AD brain, GGA3 protein levels were significantly decreased and this decrease was inversely correlated with elevations in BACE1 in AD-relevant regions, [136]. While other post-translational mechanisms may account for this observed decrease in GGA3, it is tempting to speculate that apoptotic events may play a role in the increase in BACE1 in AD brain, Indeed, elevated BACE1 activity may lead to increased Aβ levels and, given that Aβ can induce apoptosis, this could potentially trigger a vicious cycle that self-potentiates Aβ generation and cell death.

### Concluding remarks

BACE1 remains a prime drug target for inhibiting the production of Aβ. Not only does BACE1 initiate Aβ formation, but the observation that BACE1 levels are elevated in AD [97,166-170] provides a direct and compelling reason to develop therapies directed at BACE1 inhibition thus reducing Aβ and its associated toxicities. Early on, data indicated that BACE knockout mice were phenotypically normal although more recent, detailed analyses show that complete abolishment of BACE1 activity may have potentially deleterious effects. Therefore, a partial, rather than full, inhibition of this enzymatic activity may be beneficial, although the percentage of BACE1 inhibition required to significantly delay amyloid pathology and the associated cognitive changes, remains to be determined.

The physiological role of BACE1 remains to be established. However, several non-APP BACE1 substrates have been recently identified and the inclusion of NRG1 and VGSCβ subunits as putative substrates for this enzyme, indicates a potential role for BACE1 in the regulation of neuronal function. BACE1 levels are elevated under a variety of conditions, including those cellular changes evoked under the stressful conditions of cardio- and cerebrovascular disease ([97,166,173,209-215]). Indeed, evidence suggests that the BACE1 substrates identified so far may play a role in the responses to stress and/or injury, such as axonal growth and the regulation of glial cell survival (NRG1; [240]), recovery from excitotoxicity (Aβ [65]), Aβ clearance (LRP; [241]), synapse formation (APP, APLP1, APLP2; [242,243]), neuroprotection (secreted APP ectodomain; reviewed in [244]) and immune functions (PSGL-1, ST6Gal I and IL-1R2; [160,161,164]). We speculate that BACE1 may be involved in the response to stress and/or injury and that the elevation in BACE1 levels facilitates recovery after acute stress/injury. Indeed, it appears plausible that cleavage of specific BACE1 substrates is necessary for this function.

However, it may well be the case that chronic stress/injury results in pathologic BACE1 levels and deleterious amyloid formation. Indeed, chronic stress/injury could cause long-term BACE1 elevation and have harmful effects attributable to cerebral Aβ overproduction. Furthermore, we suggest that increased BACE1 activity may affect normal synaptic functioning, given that, in addition to Aβ, other BACE1 cleavage products, including specific Na_v _1s (such as Na_v_1.6) are localized to the synapse. Changes in the normal function of the synapse may result in the neurochemical deficits and behavioral abnormalities that have been reported in BACE1 transgenic models [16,18,61,70].

AD etiology remains elusive and the cause of the elevated Aβ levels observed in SAD remain to be determined. Indeed, whether increased BACE1 activity is sufficient to induce AD pathogenesis, and the initial cause (s) of the BACE1 elevation remains unknown. Several lines of evidence suggest that the observed BACE1 elevations may occur throughout the course of AD development. While our data indicates that the BACE1 elevation in AD is actively involved in AD progression and may be detected prior to the appearance of overt neurodegeneration [97], increases in BACE1 activity have also been demonstrated *in vivo *occurring in response to the induction of apoptosis; an event associated with the later stages of neurodegeneration [136]. It is plausible that different factors cause elevations in BACE1 levels and activity at different stages of the disease. The association of elevated BACE1 with overt amyloid pathology, in both Tg mouse models of AD and AD brain, indicates that Aβ may potentially cause the observed elevations in BACE1. Indeed, accumulating evidence suggests that BACE1 is a stress response protein [136,210-213,215,228,229] and Aβ is a known neurotoxin. However, while elevated Aβ levels may facilitate positive feedback loop whereby increased Aβ facilitates enhanced BACE1 activity and auto-potentiates its own production, there is accumulating data which links several well-established AD risk factors (cardiovascular diseases, stroke, ischemia and TBI), and their associated molecular changes (hypoperfusion, hypoxia, metabolic dysfunction, energy inhibition and oxidative stress), with increases in BACE1.

The molecular mechanisms governing these associations are being intensely investigated. It is apparent that BACE1 expression is tightly regulated in a complex manner at both transcriptional (HIF; [214,224] and post-transcriptional levels including translational efficiency [102], lysosomal targeting [134], and proteasomal degradation [141]. Furthermore, additional regulation of BACE1 levels may be attributed to alterations in the levels of proteins known to associate with and affect the intracellular trafficking of the enzyme (GGA3; [136]). Clearly understanding the molecular mechanisms of BACE1 elevation during SAD may facilitate the development of novel therapeutic strategies to treat, and ultimately prevent this neurodegenerative disorder.

## Abbreviations

Aβ : β-amyloid;

AD : Alzheimer's disease;

ADAM : a disintegrin and metalloprotease domain protein;

APLP : amyloid precursor-like proteins;

ApoE : apolipoprotein E;

APP : amyloid precursor protein;

BACE1 : β-site APP Cleaving Enzyme 1; 

BACE1^-/- :^ BACE1 knockout; 

CBF : cerebral blood flow;

CBH : chronic brain hypoperfusion;

CNS : central nervous system;

CRF : corticotrophin-releasing factor.

CTF : carboxyl terminal fragment;

DS : Down's syndrome; 

EGF : epidermal growth factor;

ER : endoplasmic reticulum;

FAD : familial AD; 

GGA : Golgi-localized γ-ear containing ADP ribosylation factor-binding;

GPI : glycosylphosphatidylinositol;

HIF-1 : hypoxia-inducible factor 1;

HMGCoA : hydroxymethylglutaryl-coenzyme A;

HNE : 4hydroxy-2-nonenal;

HRE : hypoxia-responsive element;

ICD : intracellular domain;

IL1β : interleukin β;

INFγ : interferon γ;

JNK : c-jun N-terminal kinases;

LRP : lipoprotein receptor-related protein;

MBP : myelin basic protein;

MCI : mild cognitive impairment;

NRG : neuregulin 1;

NSAIDS : nonsteroidal anti-inflammatory drugs;

PAR-4 : prostate apoptosis response-4;

PC : proprotein convertase;

PI3K : phospatidylinositol-3-OH kinase;

PLP : proteolipid protein;

PNS : peripheral nervous system;

PPARγ : transcriptional regulator proliferator-activated receptor γ;

PS : presenilin; 

RIP : regulated intramembrane proteolysis;

RNAi : RNA interference;

SAD : sporadic AD; 

ST6Gal1 : Beta-galactoside alpha 2,6-sialyltransferase;

TACE : TNF-α converting enzyme;

TBI : traumatic brain injury;

Tg : transgenic;

TGN : trans Golgi network;

TNFα : tumor necrosis factor α;

UTR : untranslated region;

VGSC : voltage-gated sodium channel.             
